# Pistil Smut Infection Increases Ovary Production, Seed Yield Components, and Pseudosexual Reproductive Allocation in Buffalograss

**DOI:** 10.3390/plants3040594

**Published:** 2014-12-01

**Authors:** Ambika Chandra, David R. Huff

**Affiliations:** 1 Texas AgriLife Research, Texas A&M System, 17360 Coit Road, Dallas, TX 75252, USA; E-Mail: a-chandra@tamu.edu; 2 Department of Plant Science, The Pennsylvania State University, 116 ASI Bldg., University Park, PA 16802, USA

**Keywords:** *Salmacisia*, *Buchloë*, parasitic castration, induced hermaphroditism, sexual reproductive allocation, vegetative reproductive allocation, harvest index

## Abstract

Sex expression of dioecious buffalograss [*Bouteloua dactyloides* Columbus (syn. *Buchloë dactyloides* (Nutt.) Engelm.)] is known to be environmentally stable with approximate 1:1, male to female, sex ratios. Here we show that infection by the pistil smut fungus [*Salmacisia* *buchloëana* Huff & Chandra (syn. *Tilletia buchloëana* Kellerman and Swingle)] shifts sex ratios of buffalograss to be nearly 100% phenotypically hermaphroditic. In addition, pistil smut infection decreased vegetative reproductive allocation, increased most seed yield components, and increased pseudosexual reproductive allocation in both sex forms compared to uninfected clones. In female sex forms, pistil smut infection resulted in a 26 fold increase in ovary production and a 35 fold increase in potential harvest index. In male sex forms, pistil smut infection resulted in 2.37 fold increase in floret number and over 95% of these florets contained a well-developed pistil. Although all ovaries of infected plants are filled with fungal teliospores and hence reproductively sterile, an average male-female pair of infected plants exhibited an 87 fold increase in potential harvest index compared to their uninfected clones. Acquiring an ability to mimic the effects of pistil smut infection would enhance our understanding of the flowering process in grasses and our efforts to increase seed yield of buffalograss and perhaps other grasses.

## 1. Introduction

Sexual reproduction in perennial grasses is a complicated process requiring an orchestration of coordinated gene expression while adhering to a specific sequence of developmental events. Maturation is the first step in this process whereby basal meristems become mature enough to perceive environmental stimuli, such as cold temperatures and/or daylength, which induces the transformative flowering process to begin. Under appropriate environmental conditions, the induced vegetative meristems begin to transition into a flowering apex through a process of development and elongation. Evolution molds the timing and extent of resources allocated towards the flowering process in order to maximize fitness among species. However, microbial organisms are known to pirate the flowering process for their own benefit [1,2] and in doing so may greatly alter the flowering process [3,4,5,6,7,8]. Thus, microbes provide opportunities for discovery of aberrant phenotypes in the flowering process not accounted for by currently known plant mutations [9]. As such, attention is now beginning to become focused on the deviant results caused by microbial modification of plant development as a means of enhancing our understanding and potential manipulation of the flowering process. Here we examine the morphological and resource allocation modifications caused by the biotrophic pistil smut fungus [*Salmacisia buchloëana* Huff and Chandra (syn. *Tilletia* *buchloëana* Kellerman & Swingle)] in the perennial Poaceae (Gramineae) buffalograss [*Bouteloua dactyloides* Columbus (syn. *Buchloë dactyloides* (Nutt.) Engelm.)].

Buffalograss is a stoloniferous, sod-forming grass native to the Great Plains and is considered to be the typic example of dioecious sex expression among North American grasses [10]. Sex expression of dioecious buffalograss is known to be environmentally stable with approximate 1:1, male to female, sex ratios [11,12,13]. Buffalograss exists as three chromosomal races with the diploid (2n = 2x = 20 chromosome) race containing only separate male and female dioecious sex forms while the autopolyploid (2n = 4x = 40 chromosome and 2n = 6x = 60 chromosome) races may also contain monoecious individuals and are therefore considered to be subdioecious. Buffalograss most likely evolved dioecious sex expression from a hermaphroditic ancestor [14]. Hermaphroditic flowers containing both male and female sex organs within the same floret (flower) have, on rare occasion, been observed in male spikelets of polyploid races [15]. When hermaphroditic flowers occur they are fully capable of producing viable seed (Huff [16]). In addition to sexual reproduction, buffalograss also reproduces clonally through the production of intravaginal and extravaginal branching stems that give rise to structures known as tillers and stolons, respectively. As such, buffalograss has the capability of propagating individual genotypes through space and time through vegetative reproduction.

The Basidiomycetes fungus pistil smut has long been known to induce the development of ovaries in genetically male sex forms of dioecious buffalograss [17,18]. The pistil smut fungus grows systemically within the host’s meristems and requires a host ovary to complete its life cycle. Pistil smut infection induces ovary development in male sex forms of buffalograss potentially through its ability to down-regulate the female sterility gene, *BdTs2*, that otherwise arrests pistil formation in the early stages of male floret (grass flower) development [19]. Even though no signs of the fungus have ever been observed in stamens, pistil smut infection has also been observed to enlarge the vestigial stamens of genetically female buffalograss and thus, pistil smut infection induces phenotypic hermaphroditism in both male and female sex forms of buffalograss [18,19].

Animal parasites which parasitize their animal host’s reproductive organs are often observed to divert resources from the host’s sexual reproduction process towards the “body” of the host thereby enhancing parasite survival [20]. In some cases, plant systemic fungal infection by smuts and mutualistic endophytic fungi (e.g., *Neotyphodium* spp.) have been observed to divert the energy and resources that would have been utilized for host sexual reproduction towards the vegetative growth of perennial grass hosts [21,22]. However, in other cases, plant fungal parasites have been shown to modify host floral structures to enhance their own reproductive development. One such system is the well-known example of induced hermaphroditism in white campion (*Silene latifolia* Poir. ssp. *alba* (P. Mill.) Greuter & Burdet) infected with the anther smut fungus (*Microbotryum violaceum* (Pers) G. Deml & Oberw. (syn. *Ustilago violacea* (Pers) Fuckel). The anther smut fungus infects the flowers of its host and induces the development of anthers in otherwise female plants and then sporulates only within the anthers of infected flowers [23]. Hence, anther smut’s ability to induce and sporulate within male sex organs is in sharp contrast to pistil smut’s ability to induce and sporulate within female sex organs; both are examples of induced hermaphroditism but for opposite sexes. Plants of white campion infected with the anther smut fungus have also been found to exhibit additional secondary symptoms including earlier flowering times [3], a higher number of smaller and less symmetrical flowers [3,4], delayed senescence of infected flowers [23,24], and a lower rate of plant mortality [25]. Interestingly, anther smut infection has also shown potential for altering host resource allocation in that infected plants have been observed to exhibit reduced root biomass [4].

Plants normally allocate energy and resources towards either vegetative growth, sexual reproduction, or maintenance, depending on environmental conditions and the developmental stage of their life cycle [26,27,28]. Annual plant species allocate nearly all resources towards sexual reproductive allocation after a brief vegetative growth phase (e.g., [29]). Moreover, annual grass crops like wheat, rice, and maize have been bred for thousands of years for increased sexual reproductive allocation. A classic example of plant breeders manipulating resource allocation in annual crop plants is the introduction of dwarfing genes in wheat and rice leading to the Green revolution of the 1960s [30]. Perennial plant species, on the other hand, often exhibit higher vegetative growth allocation by accumulating a greater proportion of photosynthates in leaves, stems, and roots than in seeds and typically exhibit more complicated patterns of resource allocation which may include multiple seasons of vegetative growth in order to attain a particular size before sexual reproduction begins and/or allocation towards storage organs for regrowth after surviving unfavorable growing conditions (see [28]).

Resource allocation patterns for perennial grasses are also complicated by having two modes of reproduction, namely, (1) sexual reproduction, in the traditional sense, resulting in seed that is often capable of long-term survival in soil and long distance dissemination, and (2) vegetative reproduction through intravaginal (forming tillers) and extravaginal (forming rhizomes and stolons) branching patterns [31] that perpetuate a single genotype over moderate distances of up to 0.8 km and through long periods of time approaching 1,000 years [32,33,34]. In other words, as the vegetative basal meristem of perennial grasses, called a crown, transitions into a flowering apex, the basic unit of the grass plant, known as a tiller which includes a shoot, roots, and the crown itself, will die upon completion of the flowering process (just like an annual plant). Thus, in order to be perennial, the grass’s crown must produce daughter tillers and/or lateral stems (rhizomes or stolons) prior to the transformation of the crown into the flowering structures (culm, inflorescence, spikelets, florets, stamen, and pistil) and subsequent death of the tiller. Therefore, in assessing the resource allocation in buffalograss, we need to consider both the vegetative reproductive allocation as the amount of energy and resources allocated towards its asexual reproduction through vegetative propagation as well as the sexual reproductive allocation which is the amount of energy and resources allocated towards the process of sexual reproduction.

Resources allocated for sexual reproduction may be further partitioned into male* versus* female function (sex allocation) and is theoretically governed by a trade-off hypothesis whereby gains in fitness of one sex must be greater than the cost of reducing allocations to the opposite sex for the evolution of separate sexes to occur [35]. When no such fitness gains are realized, then hermaphroditism (bisexual) is the favored evolutionary stable strategy [35]. A similar type of theoretical trade-off exists between sexual reproductive allocation and vegetative reproductive allocation. Evolution molds sexual reproductive allocation and vegetative reproductive allocation differently in different organisms depending on environmental adaptations and life history characteristics [35]. For example, grass species utilized for their high asexual vegetative reproduction, such as for forage and/or turfgrass (e.g., buffalograss), typically exhibit a low amount of sexual reproductive allocation which becomes a major limitation for commercialization because of their reduced seed yields and low seed harvest index.

In agriculture, sexual reproductive allocation is commonly measured as seed yield or as harvest index. Both seed yield and harvest index are capable of being described by a multiplicative formula of various seed yield components, for example:

(1) $$\text{Seed}\;\text{Yield}=\text{number}\;\text{of}\;\text{inflorescences}{\text{ plant}}^{-1}\times \text{number}\;\text{of}\;\text{branches}{\text{ inflorescence}}^{-1}\times \text{number}\;\text{of}\;\text{seeds}{\text{ branches}}^{-1}\times \text{weight}{\text{ seed}}^{-1}$$

(2) Harvest Index=seed yield/total plant biomass

The ideal agronomic crop plant that breeders attempt to realize through their genetic manipulations typically have improved seed yield as a main goal. Even in perennial grasses utilized as forage or turfgrass, where vegetative biomass and recuperative capacity are valued traits, maintaining or improving seed yield is also important for commercialization [36]. However, achieving gains in seed yield in crop plants is complicated by the polygenic nature of the trait, by trade-offs among the individual seed yield components, by trade-offs among vegetative reproductive allocation and sexual reproductive allocation (as is often the case in perennial grasses), and by limitations in overall photosynthesis and total resource acquisition. Furthermore, breeding for high seed yielding cultivars requires a long-term commitment as well as overcoming the various challenges posed by a variety of biotic and abiotic stresses. One of the greatest threats to seed yield productivity in grain crops is phytopathic microorganisms, especially those caused by fungi [37], for example the parasitic smut and bunt diseases of the genus *Tilletia* [38], a sister clade of *Salmacisia* [18].

In the present study, we measured the effects of pistil smut infection on the morphology and resource allocation of diploid buffalograss. Smuts are economically important due to their devastating impact on food grain crops worldwide and, as such, nearly all efforts are focused on the prevention, control, and embargo identification of smuts in world agriculture [38]. However, the buffalograss-pistil smut provides a non-threatening model system in which to explore the ability of smut infection to regulate its host sex expression, meristem determinacy, and resource allocation in order to enhance our understanding of the flowering process in grasses. Here we show that the parasitic pistil smut fungus demonstrates a remarkable ability to increase the overall sexual reproductive allocation of buffalograss by systematically enhancing various seed yield components of both male and female sex forms. It is important to note here that plants infected with the pistil smut fungus do not produce viable seed but rather the mature ovaries of infected plants are filled with fungal teliospores and are thus, reproductively sterile. In order to make this important distinction more clear, we have elected to use the term “pseudosexual reproductive allocation” when referring to sexual resource allocation involving infected plants. If pistil smut’s ability to manipulate host pseudosexual reproductive allocation were better understood and capable of being realized without the fungus, it may actually benefit agriculture by improving the harvest index of grain crops in general and specifically in perennial grain crops whose low seed yields have been problematic for commercialization [39].

## 2. Results and Discussion

### 2.1. Pistil Smut Fungus Life Cycle and Secondary Effects of Pistil Smut Infection

**Figure 1:**
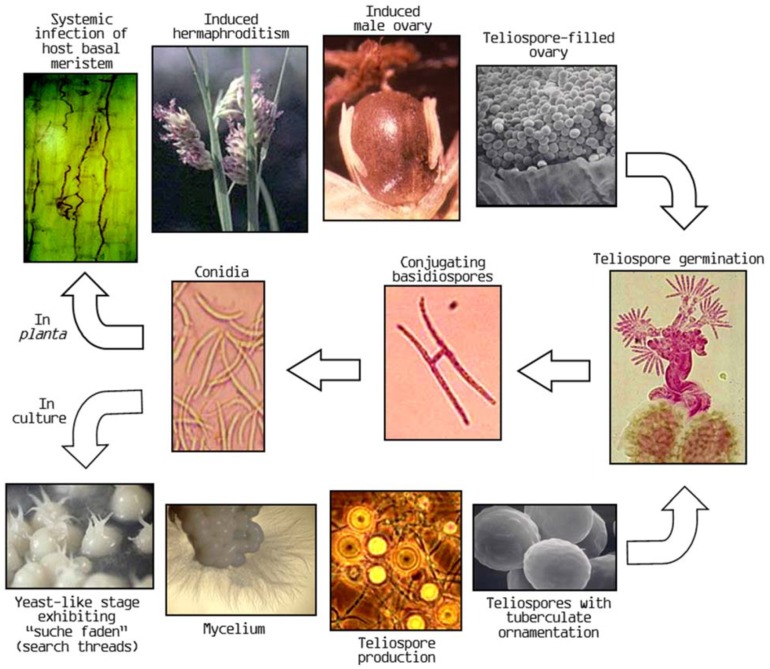
Life cycle of the pistil smut fungus (*Salmacisia buchloëana* H&C) in relation to its host buffalograss and in culture.

The life cycle of pistil smut fungus begins as a germinating diploid teliospore in soil (Figure 1). Teliospore germination produces a promycelium that bears finger-like primary basidiospores which often require conjugation in order to be capable of growing a mycelia dikaryon that produces secondary sporidia. It is these secondary spordia that produce infection hyphae which penetrate the basal meristem tissue of the buffalograss host. Once inside its host, the pistil smut fungus grows systemically within the host’s vegetative meristems (crowns). Prior to the flowering process, infection by the pistil smut fungus is virtually symptomless. However, when the crown begins to differentiate from a vegetative meristem into a flowering apex, the pistil smut fungus interacts with the developmental process to induce female sex organs in its unisexual male hosts resulting in its namesake symptom of purple feathery pistillate stigmas exerting from “male” florets. The fungus completes its reproductive cycle by filling the induced ovaries, as well as those of infected female sex forms, with copious amounts of diploid teliospores. In water agar culture, the secondary sporidia grow into small mounds with a “yeast-like” consistency and display structures known as “such faden”, literally “search threads” in German. Eventually, cultures begin to display mycelium which finally produces diploid teliospores on hyphal tips.

**Figure 2:**
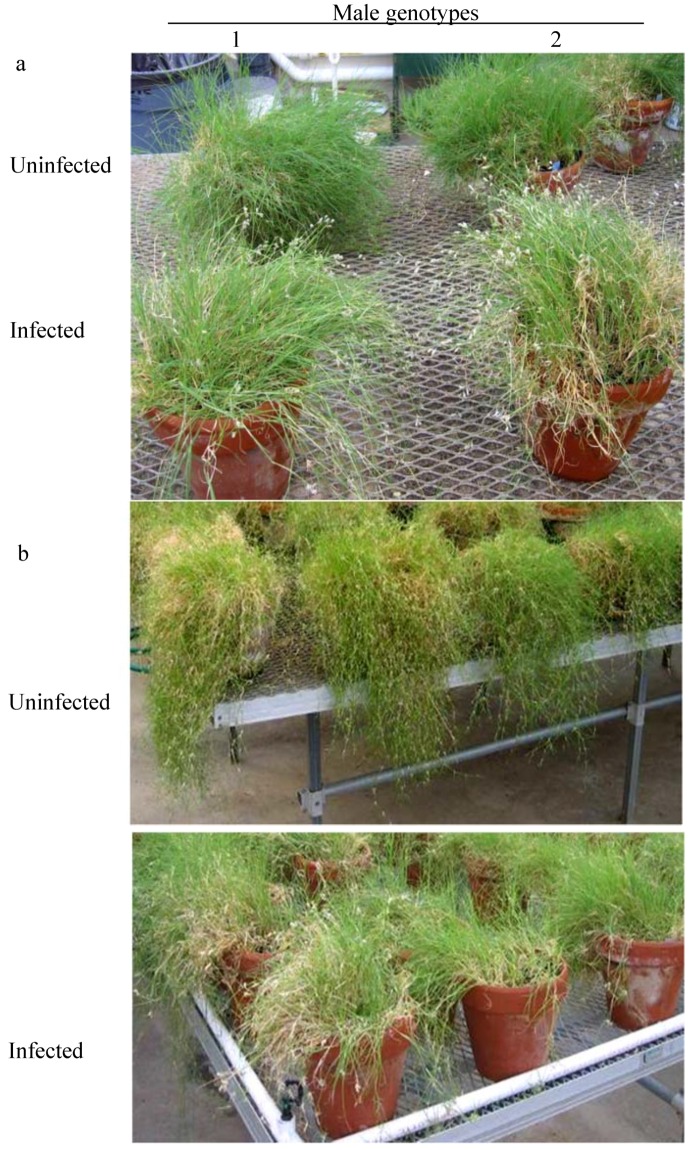
Effects of pistil smut infection on resource allocation in buffalograss. (**a**) infected male plants were observed to flower more profusely compared to uninfected clones of the same genotype. (**b**) uninfected male and female genotypes appeared to produce more vegetative stolons compared to infected genotypes.

**Figure 3:**
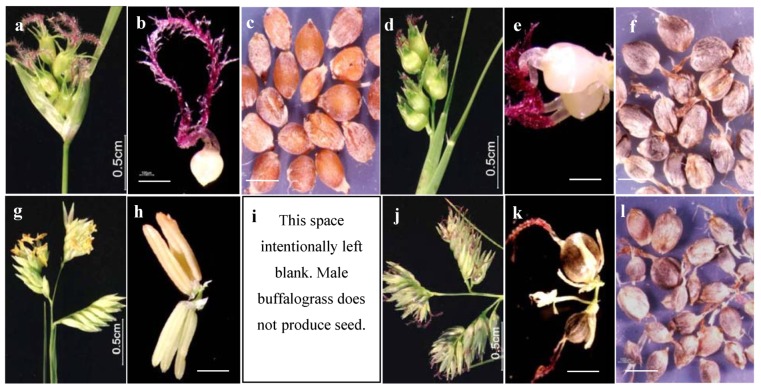
Developmental changes in inflorescence architecture and ovary development for buffalograss infected by the pistil smut fungus. (**a**) inflorescence of uninfected female buffalograss. (**b**) a spikelet of uninfected female buffalograss contains a single pistil (pictured) with fully developed stigmas, style, and ovary. (**c**) seed (caryopses) produced by uninfected female buffalograss. (**d**) inflorescence of infected female buffalograss. (**e**) a spikelet of infected female buffalograss contains two pistils (pictured) each with fully developed stigmas, style, ovary, and hypertrophied stamens. (**f**) smut balls, not seed, are formed in florets of infected female as ovaries become filled with fungal teliospores. (**g**) inflorescence of uninfected male buffalograss. (**h**) a spikelet of uninfected male buffalograss contains two stamens (pictured) each with fully developed anthers and filaments. (**i**) this space was intentionally left blank as no seed is produced by uninfected male buffalograss. (**j**) inflorescence of infected male buffalograss displays the presence of purple feathery stigmas. (**k**) a spikelet of infected male buffalograss contains three pistils (pictured) each with fully developed (or developing) stigmas, style, ovary, and underdeveloped stamens. (**l**) smut balls form in florets of infected male buffalograss as induced ovaries become filled with fungal teliospores. Bar = 0.25mm unless otherwise indicated.

During our previous investigations of the buffalograss-pistil smut system, we began to notice that infected plants tended to flower more profusely and produce fewer stolons than uninfected plants (Figure 2a,b). In addition, pistil smut infected plants appeared to exhibit a crowding of florets on their inflorescences (Figure 3g* vs.* Figure 3j). Thus, infected plants appeared to exhibit higher levels of pseudosexual resource allocation and lower levels of vegetative resource allocation compared to uninfected plants. In order to determine if any additional secondary effects of pistil smut infection were present in buffalograss, and if any such differences were equally apparent in both male and female sex forms, we designed the present study to enable a side-by-side comparison of clonally propagated plants from individual genotypes of buffalograss which were either infected or uninfected with the pistil smut fungus.

### 2.2. Biomass Accumulation and Resource Partitioning

Pistil smut was found to substantially alter biomass accumulation and resource partitioning in buffalograss during the present study (Table 1). Total biomass per plant was reduced 34% (−1.52 fold decrease) in female sex forms and 36% (−1.55 fold decrease) in male sex forms. Vegetative biomass was impacted the most as a result of infection with an observed −1.83 reduction in female sex forms and a −1.79 reduction in male sex forms. However, pistil smut actually increased the sexual biomass of infected plants. The sexual biomass of females was increased 4.55 fold (455% increase) in female sex forms and 4.88 fold (488% increase) in male sex forms as a result of infection. Taken together, these results have an additive effect on significantly increasing the overall sexual reproductive allocation while significantly decreasing vegetative reproductive allocation in infected plants (Table 1). The previously defined pseudosexual reproductive allocation of infected plants was increased 7.21 fold for female plants and 5.22 fold for male plants compared to their uninfected clones while, vegetative reproductive allocation was reduced by −1.21 fold and −1.16 fold for female and male sex forms, respectively. These results suggest that pistil smut infection appears to have reprogrammed plants to divert more energy and resources towards the flowering and sexual reproductive processes in a proportion greater than the reduction of resources diverted from vegetative reproductive allocation, compared to their genetically identical uninfected counterparts.

**Table 1:** Mean resource partitioning for female and male genotypes of buffalograss that were either infected or uninfected with the pistil smut fungus. For each genotype, two replicate clonal plants were infected with pistil smut and two remained uninfected. Means are the clonal averages across genotypes from greenhouse grown plants. Mean differences between infected and uninfected clones were tested for significance using Student’s paired t-test. Standard error of mean = (SEM).

	Biomass plant^−1^			Resource allocation plant^−1^	
	Total ^1^ (g)	Vegetative ^2^ (g)	Sexual ^3^ (g)	Vegetative (VRA) ^4^	Sexual (SRA) ^5^
Female (N = 23)					
Infected	85.0	68.5	16.51	0.803	0.197
Uninfected	128.9	125.2	3.63	0.973	0.027
Avg. difference	−43.9 ***	−56.7 ***	12.89 ***	−0.170 ***	0.170 ***
(SEM)	(5.09)	(4.99)	(2.20)	(0.023)	(0.023)
Fold difference ^6 ^	−1.52	−1.83	4.55	−1.21	7.21
Male (N = 31)					
Infected	77.3	64.9	12.46	0.833	0.167
Uninfected	120.0	116.2	3.78	0.968	0.032
Avg. difference	−42.6 ***	−51.3 ***	8.67 ***	−0.135 ***	0.135 ***
(SEM)	(4.86)	(5.13)	(1.17)	(0.016)	(0.016)
Fold difference ^6^	−1.55	−1.79	3.29	−1.16	5.22

*** Significant at the 0.1% level of probability; ^1^ Total plant biomass = vegetative biomass + sexual biomass; ^2^ Vegetative biomass = dry weight of all nonflowering vegetative shoots and stems; ^3^ Sexual biomass = dry weight of all flowering culms; ^4^ VRA, Vegetative reproductive allocation = vegetative biomass/total plant biomass; ^5^ SRA, Sexual reproductive allocation = sexual biomass/total plant biomass. In addition, termed as pseudosexual reproductive allocation in the text when specifically referring to plants infected with the pistil smut fungus; ^6^ Increase (+) or decrease (−) of clonal average for infected clones compared to uninfected clones were measured as fold difference,* i.e.*, (infected/uninfected). Negative fold differences (fractional values) were transformed by taking their inverse to attain whole negative numbers.

Determining the optimal strategy of resource allocation in buffalograss is complicated by its perennial nature which necessitates survival during unfavorable growing conditions (ex. freezing temperatures and prolonged drought), its capacity for simultaneous vegetative (clonal) reproduction and sexual (seed) reproduction, and its capacity for longevity which likely is of great age. Sexual reproductive allocation is normally low for buffalograss ranging from 1% to 3% of total plant biomass while vegetative reproductive allocation typically ranging from 97% to 99% [13]. The vegetative biomass of buffalograss is cumulatively produced by two different types of branching patterns known as intravaginal and extravaginal branching (see [31]). The relative proportion of tillers (intravaginal branching) compared to stolons (extravaginal branching) in buffalograss on a per plant dry weight basis has been shown to range from 14%–23% for tillers and 75%–85% for stolons [13]. However, as both tillers and stolons are forms of vegetative propagation that achieve perennially of the genotype through space and time [33,34], we calculated vegetative reproductive allocation as the combined dry weight of all non-flowering tillers and stolons divided by the total aboveground dry weight.

Plants have evolved a coordinated movement of resources collected from photosynthesis in the leaves and mineral acquisition and uptake in the roots (sources) towards numerous vegetative and floral growing point meristems (sinks). This orchestrated movement of resources from source to sink is flexible and modulated throughout a plant’s life cycle and seasonal growth and has also been molded by evolution to fit a defined set of life-history characteristics. However, there are examples of systemic fungi redirecting metabolic flow of the host [40,41], modifying host resource partitioning towards enhanced vegetative reproductive allocation [42], and reducing root biomass in favor of a higher number of smaller sized flowers [4] compared to uninfected plants. Similarly, the pistil smut fungus appears fully capable of reprogramming the metabolic flow of buffalograss resource allocation, by altering sink strength and sink location to enhance its host’s pseudosexual reproductive allocation, and thereby enhancing its own survival and reproduction.

### 2.3. Seed Yield Components

In order to ascertain the details of this extraordinary increase of pseudosexual reproductive output in buffalograss by the pistil smut fungus, data was collected for various components of seed yield and harvest index (Table 2). Number of inflorescences per plant, spikes per inflorescence, florets per spikelet, and estimated florets per plant were all significantly increased in genotypes of both male and female sex forms upon pistil smut infection. Number of spikelets per spike was also significantly increased in females upon infection, however, spikelets per spike was significantly decreased in infected males compared to uninfected males. Of all seed yield component parameters examined, number of inflorescences per plant was proportionately increased the most by pistil smut infection confirming our earlier observations (Figure 2a). Number of inflorescences was increased by 6.7 fold in females and 2.37 fold in males (Table 2).

The next most influential seed yield component was florets per spikelet which exhibited a 2.0 fold increase in females and a 1.39 fold increase in males as a result of infection (Table 2). Typically, uninfected female sex forms of buffalograss bear only a single floret per spikelet and uninfected males bear two florets per spikelet, however, pistil smut infection was found to induce the development of an additional floret per spikelet for both sex forms (Figure 3b *vs.* Figure 3e and Figure 3h *vs.* Figure 3k; Table 2). This observation confirms Engelmann’s [43] statement that florets of male buffalograss may contain a third floret per spikelet and explains the line drawing by Kellerman and Swingle [17] depicting an infected male spikelet with three florets. Grasses have a complex inflorescence structure and thus the flowering process involves multiple types of meristems [44]. During the reprogramming of the flowering process, the pistil smut fungus appears capable of altering the determinacy of buffalograss meristems, either positively or negatively. For example, we found that meristems that normally would have remained vegetative (indeterminate) become transitioned into flowering apices (determinate) and result in a greater number of inflorescences on infected plants. Conversely, floral meristems that would normally produce a limited number of florets (determinate: one floret per female spikelet and two florets per male spikelet) continue to grow and develop in infected plants to produce an additional floret (*i.e.*, more indeterminate) for each sex form.

**Table 2:** Measured and estimated means of seed yield components for female and male genotypes of buffalograss that were either infected or uninfected with the pistil smut fungus. For each genotype, two replicate clonal plants were infected with pistil smut and two remained uninfected. Means are the clonal averages across genotypes from greenhouse grown plants. Mean differences between infected and uninfected clones were tested for significance using Student’s paired t-test. Standard error of mean = (SEM).

	Inflorescences plant^−1^	Spikes inflor.^−1^	Spikelets spike^−1^	Florets spikelet^−1^	Estimated florets plant^−1 1^
Female					
Genotypes (N)	23	20	20	20	20
Infected	141.9	3.58	4.84	2.00	5,683
Uninfected	21.2	3.16	2.95	1.00	219
Avg. difference	120.7 ***	0.412 **	1.89 ***	1.00 ***	5,464 ***
(SEM)	(14.00)	(0.150)	(0.473)	(0.079)	(1,002)
Fold difference ^2^	6.70	1.13	1.64	2.00	25.98
Male					
Genotypes (N)	31	28	28	28	28
Infected	127.4	3.41	12.22	2.83	15,809
Uninfected	53.8	2.86	13.20	2.00	4,529
Avg. difference	73.6 ***	0.55 ***	−0.98 **	0.83 ***	11,280 ***
(SEM)	(10.56)	(0.102)	(0.380)	(0.055)	(1,379)
Fold difference ^2^	2.37	1.19	−1.08	1.39	2.37

**, *** Significant at the 1%, and 0.1% level of probability, respectively; ^1^ Estimated florets per plant = (inflorescences/plant) × (spikes per inflorescence) × (spikelets/spike) × (florets/spikelet); ^2^ Increase (+) or decrease (−) of clonal average for infected clones compared to uninfected clones were measured as fold difference,* i.e.*, (infected/uninfected). Negative fold differences (fractional values) were transformed by taking their inverse to attain negative whole numbers.

There are few comparable fungal-plant host systems that exhibit such dramatic alterations of both seed yield components and resources allocation to the extent we have observed in the pistil smut-buffalograss system. One such system is the well-known example of induced hermaphroditism in white campion infected with the anther smut fungus which not only induces anthers in female sex forms but has also been observed to delay senescence of infected flowers [23,24], increase the number of flowers per plant (however these flowers are of smaller size and less symmetrical than uninfected flowers) [3,4], reduce root biomass allocation presumably for enhanced pseudosexual reproductive allocation [4], and increase life expectancy over a five year period [25]. Other examples include the common bunt (*Tilletia caries* (D.C.) Tul.) and dwarf bunt (*Tilletia controversa* Kühn) diseases of wheat (*Triticum aestivum* L.). Wheat normally displays 2 to 3 florets per spikelet however plants infected with common bunt usually have 3 to 5 florets per spikelet while plants infected with dwarf bunt commonly have 5 to 10 florets per spikelet [5,6]. Both common and dwarf bunt belong to genus *Tilletia* which, as a sister genus to pistil smut, suggests that the ability to induce additional florets per spikelet may be a homologous trait among these three species.

### 2.4. Potential Seed Yield and Harvest Index

One caveat that deserves reiterating at this point is that the pistil smut infected plants do not produce viable seed but rather the mature ovaries (induced or not) of all infected florets (male or female) are filled with fungal teliospores and are thus, reproductively sterile (Figure 3c *vs.* Figure 3f or Figure 3l). Here we assume that the energetic costs to the infected plants filling their ovaries with fungal teliospores is roughly equivalent to that of uninfected plants filling their fertilized ovaries with starchy endosperm during seed formation. Under this assumption, we refer to the fungal-induced increase in estimated buffalograss seed yield as a “potential” seed yield increase. In addition, the weight per seed component of seed yield can only be calculated for uninfected female plants whereas for infected plants of both sexes this component is represented as weight per smut ball (*i.e.*, a teliospore filled ovary) (Table 3).

Overall, pistil smut infection induced a 25.98 fold increase in the estimated number of florets per plant in females and each of these florets examined contained a pistil ovary (Table 3). In male sex forms, pistil smut infection induced a 2.37 fold increase in the estimated number of florets per plant and over 95% of these florets (784 out of 827 florets examined) contained a well-developed fungal induced pistil ovary, while the remaining 5% (43 florets) seemed to have escaped infection and appeared to function as normal pollen-producing male florets (Table 3).

We found that average potential seed yield per uninfected female plant (0.171 g) was substantially less than the average potential smut ball yield of infected female plants (3.66 g), resulting in a 21.37 fold increase due to infection (Table 3). Given our assumption that the energetic costs are roughly equivalent between smut ball and seed production, the potential for seed yield increase in buffalograss would be substantial if breeders/geneticists were able to manipulate buffalograss in the same way as pistil smut. The average estimated weight of smut balls produced per infected male plant (6.01 g) was over 1.5 times the weight of infected female plants (3.66 g), however, the estimated average number of induced male ovaries (15,019) was 2.5 times greater than that of infected female plants (5,683) and thus male smut balls were smaller and weighed less than female smut balls (Figure 3l *vs.* Figure 3f; Table 3). It would appear that either buffalograss physiology limits the amount of ovary filling, given the large number of ovaries produced on infected male plants (*i.e.*, a tradeoff between seed number and seed size), or that induced male ovaries are inherently smaller in size than female ovaries.

Finally, when the effects of fungal infection are applied across all seed yield components, including the equivalency of seed weight* vs.* smut ball weight, and indexed based on total aboveground plant dry weight, the result is that pistil smut infection increased estimated potential harvest index by 35.15 fold in female sex forms (Table 3).

The molecular genetic basis underlying the flowering process in grasses lags behind that of the eudicots but progress is being made. Attempts to reconcile the eudicot ABC model of flowering with known grass mutants is one attempt to better understand the flowering process in grasses [31]. In addition, research in meristem transitioning and differentiation is being conducted to help better understand the flowering process in agronomically important grasses like maize and rice [45,46]. In addition, as our knowledge of gene regulatory processes broadens, microRNAs are now being identified as having a role in regulating phase transition, floral meristem determinacy, and sex determination of grasses [47,48]. Clearly, the extent to which the pistil smut fungus alters buffalograss meristem fate, sex determination, and resource partitioning must involve numerous genes, biochemical pathways, and hormonal cross-talk. Future research to discover the genetic basis of pistil smut’s regulatory control over buffalograss flower development and resource allocation processes would likely benefit from a large scale RNA sequence and expression analysis, similar to those techniques utilized for examining head smut (*Sporisorium reilianum* Küth) infection of maize which transforms sexual reproductive tissue into vegetative tissue, a process known as phyllody [7] or floral induced mimicry caused by the rust fungus *Puccinia monica* Arth. in a member of the mustard family known as Drummond’s rockcress (*Boechera stricta* (Graham) Al-Shehbaz) [8].

**Table 3:** Potential seed yield and potential harvest index for female and male genotypes of buffalograss that were either infected or uninfected with the pistil smut fungus. For each genotype, two replicate clonal plants were infected with pistil smut and two remained uninfected. Means are the clonal averages across genotypes from greenhouse grown plants. Uninfected females yield seed (endosperm filled caryopsis) while infected males and females yield smut balls (teliospore filled ovaries). Mean differences between infected and uninfected clones were tested for significance using Student’s paired t-test. Standard error of mean = (SEM).

	Estimated ovaries plant^−1^ ^1^	Weight of 100 seeds or smut balls (sb) ^2^ (mg)	Potential seed or smut ball yield plant^−1^ ^3^ (g)	Potential harvest index plant^−1^ ^4^ (%)
Female				
Genotypes (N)	20	6	20	20
Infected	5683	64.4 (sb)	3.660 (sb)	4.697
Uninfected	219	78.3 (seed)	0.171 (seed)	0.134
Avg. difference	5464 ***	−13.9 **	3.489 ***	4.563 ***
(SEM)	(1002)	(3.50)	(0.647)	(1.006)
Fold difference ^5^	25.98	−1.22	21.37	35.15
Male				
Genotypes (N)	28	6	28	28
Infected	15019	40.0 (sb)	6.01 (sb)	8.19 (sb)
Uninfected	0	0	0	0
Avg. difference	-	-	-	-
(SEM)	-	-	-	-
Fold difference ^5^	N/A	N/A	N/A	N/A

**, *** Significant at the 1%, and 0.1% level of probability, respectively; N/A—not applicable.; ^1^ All female florets examined contained an ovary whereas, on average, only 95% of infected male florets contained a fungal induced ovary; ^2^ Actual data was collected for a subset of genotypes only; ^3^ Seed or smut ball yield = (ovaries/plant) × (average weight of a seed or smut ball); ^4^ Estimated harvest index = seed or smut ball yield / total plant biomass.; ^5^ Increase (+) or decrease (−) of clonal average for infected clones compared to uninfected clones were measured as fold difference,* i.e.*, (infected/non-infected). Negative fold differences (fractional values) were transformed by taking their inverse to attain negative whole numbers.

### 2.5. Potential Seed Yield and Harvest Index for a Male-Female Pair of Buffalograss

It is not possible to calculate an equivalent amount of increase in seed yield or harvest index as a result of infection for male sex forms because uninfected male buffalograss plants do not produce a harvestable sexual biomass. However, the estimated harvest index of an average pair of male and female sex forms is comparable between infected and uninfected plants. Thus, the estimated seed yield for a pair of uninfected female and male plants would remain at 0.171 g while that of an infected male-female pair would be 9.67 g, representing an estimated 56 fold increase in potential seed yield and an 87 fold increase in potential harvest index (Table 4).

**Table 4:** Potential seed yield and potential harvest index (yield/total biomass) for pairs of male and female buffalograss either infected or uninfected with the pistil smut fungus. Uninfected females yield seed (endosperm filled caryopses) while infected males and females yield smut balls (teliospore filled ovaries).

Male + Female pair	Potential seed or smut ball yield pair^−1^ ^1^ (g)	Potential harvest index pair^−1^ ^2^ (%)
Uninfected (Seed)	0.171	0.069
Infected (Smut balls)	9.670	6.022
Fold difference ^3^	56.550	86.994

^1^ Seed or smut ball yield = (ovaries/plant) × (average weight of a seed or smut ball); ^2^ Harvest index was calculated as the combined average pair of male and female buffalograss; ^3^ Increase (+) or decrease (−) of clonal average for infected clones compared to uninfected clones were measured as fold difference,* i.e.*, (infected/non-infected).

Our results show that the intricate regulatory control of buffalograss growth and development by the pistil smut fungus seems to primarily influence resource partitioning, phenotypic sex determination, and meristem determinancy,* i.e.*, the determinacy of vegetative meristems (transition from non-flowering to flowering) and floral meristems (developing an extra flower per spikelet), resulting in enhanced host pseudosexual reproductive allocation and a potential for greatly increased seed yield and harvest index within infected plants. If such an increase in harvest index were capable of being realized by plant breeders, in the absence of the fungus, it would certainly represent a substantial agronomic improvement. Clearly, increasing our understanding of how pistil smut is able to manipulate its host in such dramatic fashion may help unravel the complex flowering and developmental process of grass inflorescences and potentially improve our ability to increase seed yields in perennial grain, forage, and biomass crops whose low seed yields have been problematic for commercialization [39].

## 3. Experimental Section

### 3.1. Isolation and Culture of Pistil Smut

The pistil smut fungus used in this study was a single source originally collected from an individual male buffalograss plant growing in a short grass prairie located in Kingfisher County, Oklahoma in 1986 [49]. Teliospore filled ovaries were collected and surface sterilized in 70% ethanol 1 min followed by 0.25% NaClO (5% v/v commercial bleach) 3 min before soaking in distilled water 2 d. Teliospore germination was performed on 2% water agar at 25 °C under 8 h light and aseptic conditions.

### 3.2. Plant Material

In November of 2004, sixty seeds of Mexican diploid (2n = 20) buffalograss (population Mex-A) [50] were germinated on moistened paper disc and 54 seedlings (90% germination) and transplanted into plastic pots (15.25 cm diameter) containing potting soil (Promix^®^, Premier Horticulture Inc., Quakertown, PA, USA), and grown in the greenhouse (26 °C day/21 °C night) under natural day length conditions. At the 4 to 6 tiller stage (approximately three weeks of growth), each genotype was vegetatively propagated into four clonal replicate plants, making of total of 216 clonal plants.

### 3.3. Host Infection by Pistil Smut

Two of the clonal replicate plants of each genotype were left uninfected and two were inoculated by embedding teliospores of the pistil smut fungus into the soil surface close to the base of vegetative shoots. All plants were saturated with water and kept sealed in clear plastic bags in order to maintain high humidity conditions, for approximately 6 weeks allowing teliospores to germinate and the fungus to enter the infected clones and to serve as a mock infection for the uninfected clones. After 4 months of growth, each clonal plant was transplanted into 30.5 cm clay pots and grown in the greenhouse (26 °C day/21 °C night) under natural day-length conditions to study the effects of pistil smut infection. The greenhouse experiments were performed as a randomized complete block design with two blocks. One pair of infected and uninfected clones for each of the 54 genotypes was present in each block.

### 3.4. Resource Partitioning Analysis

After a total of 19 months of growth, all biomass from the resulting 23 female genotypes (92 plants) and 31 male genotypes (124 plants) infected and uninfected with pistil smut were harvested 3 cm above the potting soil surface and separated into sexual reproductive biomass (all flowering shoots,* i.e.*, culms and inflorescences) and vegetative biomass (all non-flowering shoots and stolons). Harvested plant material were placed in brown paper bags, air-dried in the greenhouse for two weeks and then received a final drying for 24 h at 55 °C and weighed. Student’s paired t-test (Minitab^®^ release 14.2 [51]) was used to analyze the differences in biomass accumulation, vegetative reproductive allocation, and sexual reproductive allocation (also termed pseudosexual reproductive allocation in the case of infected plants) between paired samples of infected and uninfected genotypic clones.

### 3.5. Analysis of Seed Yield Components

Data on seed yield components was collected from the three visually tallest inflorescences for the 23 female and 31 male genotypes infected and uninfected with pistil smut. Paired samples of infected and uninfected clones served as replicates for each genotype and the effects were averaged across genotypes within each sex form. Student’s paired t-test was conducted using Minitab [51] to analyze the mean differences for measured seed yield components between the paired samples of infected and uninfected genotypic clones.

## 4. Conclusions

Pistil smut is a biotrophic, systemic fungus that, in this study, has been shown to dramatically alter plant meristem fate, sex determination, and resource partitioning, all of which accentuates the pseudosexual reproductive output of its host buffalograss in order to further its own survival. To our knowledge, pistil smut is one of the few fungal parasites that actually increase the potential seed yield of its host. Further studies are needed to ascertain the regulatory role pistil smut has on its host’s genes and hormonal interactions. We believe such studies may provide an opportunity to discover unique regulatory processes not accounted for in known plant mutations. Furthermore, if geneticists and breeders were able to achieve the effects of pistil smut without the fungus, we believe it would be possible to significantly improve the seed yield and harvest index of perennial grasses for the betterment of the human race.

## References

[1] Gorischek A.M., Afkhami M.E., Seifert E.K., Rudgers J.A. (2013). Fungal symbionts as manipulators of plant reproductive biology. Am. Nat..

[2] Gundel P.E., Garibaldi L.A., Martínez-Ghersa M.A., Ghersa C.M. (2012). Trade-off between seed number and weight: Influence of a grass—Endophyte symbiosis. Basic Appl. Ecol..

[3] Shykoff J.A., Kaltz O. (1997). Effects of anther smut *Microbotryum violaceum* on host life-history patterns in *Silene latifolia* (Caryophyllaceae). Int. J. Plant Sci..

[4] Shykoff J.A., Kaltz O. (1998). Phenotypic changes in host plants diseased by *Microbotryum violaceum*: Parasite manipulation, side effects, and trade-offs. Int. J. Plant Sci..

[5] Purdy L.H., Kendrick E.L., Hoffman J.A., Holton C.S. (1963). Dwarf bunt of wheat. Annu. Rev. Microbiol..

[6] Trione E.J., Stockwell V.O., Latham C.J. (1989). Floret development and teliospore production in bunt-infected wheat, in planta and in cultured spikelets. Phytopathology.

[7] Ghareeb H., Becker A., Iven T., Feussner I., Schirawski J. (2011). *Sporisorium reilianum* infection changes inflorescence and branching architectures of maize. Plant Physiol..

[8] Cano L.M., Raffaele S., Haugen R.H., Saunders D.G.O., Leonelli L., MacLean D., Hogenhout S.A., Kamoun S. (2013). Major transcriptome reprogramming underlies floral mimicry induced by the rust fungus *Puccinia monoica* in *Boechera stricta*. PLoS One.

[9] Wei W., Davis R.E., Nuss D.L., Zhao Y. (2013). Phytoplasmal infection derails genetically preprogrammed meristem fate and alters plant architecture. Proc. Natl. Acad. Sci. USA.

[10] Hitchcock A.S., Chase A. (1950). Manual of the Grasses of the United States.

[11] Huff D.R. (1992). Sex ratios and inheritance of anther and stigma color in diploid buffalograss. Crop Sci..

[12] Huff D.R., Wu L. (1992). Distribution and inheritance of inconstant sex forms in natural populations of dioecious buffalograss (*Buchloë dactyloides*). Am. J. Bot..

[13] Quinn J.A., Engel J.L. (1986). Life-history strategies and sex ratios for a cultivar and a wild population of *Buchloe dactyloides* (Gramineae). Amer. J. Bot..

[14] Kinney M.S., Columbus T., Friar E.A. (2007). Dicliny in *Bouteloua* (Poaceae: Chloridoideae): implications for evolution of dioecy. Aliso.

[15] Wenger L.E. (1940). Inflorescence variations in buffalo grass, *Buchloe dactyloides*. J. Am. Soc. Agron..

[16] Huff D.R. (1984). Personal Observation.

[17] Kellerman W.A., Swingle W.T. (1889). New species of Kansas fungi. J. Mycol..

[18] Chandra A., Huff D.R. (2008). Salmacisia, a new genus of Tilletiales: Reclassification of *Tilletia buchloeana* causing induced hermaphroditism in buffalograss. Mycologia.

[19] Chandra A., Huff D.R. (2010). A fungal parasite regulates a putative female-suppressor gene homologous to maize Tasselseed2 and causes induced hermaphroditism in male buffalograss. Mol. Plant Microbe Interact..

[20] Baudoin M. (1975). Host castration as a parasitic strategy. Evolution.

[21] Clay K. (1991). Parasitic castration of plants by fungi. Trends Ecol. Evol..

[22] Kover P.X. (2000). Effects of parasitic castration on plant resource allocation. Oecologia.

[23] Uchida W., Matsunaga S., Sugiyama R., Kazama Y., Kawano S. (2003). Morphological development of anthers induced by the dimorphic smut fungus *Microbotryum violaceum* in female flowers of the dioecious plant *Silene latifolia*. Planta.

[24] Alexander H., Antonovics J. (1988). Disease spread and population dynamics of anther-smut infection of *Silene alba* caused by the fungus *Ustilago violacea*. J. Ecol..

[25] Buono L., López-Villavicencio M., Shykoff J.A., Snirc A., Giraud T. (2014). Influence of multiple infection and relatedness on virulence: Disease dynamics in an experimental plant population and its castrating parasite. PLoS One.

[26] Wilson M.F. (1983). Plant Reproductive Ecology.

[27] Plant Resource Allocation. http://www.sciencedirect.com/science/book/9780120834907.

[28] Mironchenko A., Kozłowski J. (2014). Optimal allocation patterns and optimal seed mass of a perennial plant. J. Theor. Biol..

[29] Law R. (1979). The cost of reproduction in annual meadow grass. Am. Nat..

[30] Borlaug N.E., Narvaez I., Aresvik O., Anderson R.G. (1969). Green revolution yields a golden harvest. Columbia J. World Bus..

[31] Herben T., Krahulec F., Hadincova V., Kovarova M., Skalova H. (1994). Morphological constraints of shoot demography of a clonal plant: Extra- and intravaginal tillers of *Festuca rubra*. Plant Species Biol..

[32] Liston A., Wilson B.L., Robinson W.A., Doescher P.S., Harris N.R., Svejcar T. (2003). The relative importance of sexual reproduction* versus* clonal spread in an aridland bunchgrass. Oecologia.

[33] Harberd D.J. (1961). Observations on population structure and longevity of *Festuca rubra* L. New Phytol..

[34] Harberd D.J. (1962). Some observations on natural clones in *Festuca ovina*. New Phytol..

[35] Charnov E.L. (1982). The Theory of Sex Allocation.

[36] Huff D.R., Boller B., Posselt U.K., Veronesi F. (2010). Bluegrasses. Handbook of Plant Breeding: Fodder Crops and Amenity Grasses.

[37] Reynold M.P., Borlaug N.E. (2006). Impacts of breeding on international collaborative wheat improvement. J. Agric. Sci..

[38] Mathre D.E. (1996). Dwarf bunt: Politics, identification, and biology. Annu. Rev. Phytopathol..

[39] DeHaan L.R., van Tassel D.L., Cox T.S. (2006). Perennial grain crops: A synthesis of ecology and plant breeding. Renew. Agric. Food Syst..

[40] Malinowski D.P., Belesky D.P. (2000). Adaptations of endophyte-infected cool-season grasses to environmental stresses: Mechanisms of drought and mineral stress tolerance. Crop Sci..

[41] Parker D., Beckmann M., Zubair H., Enot D.P., Caracuel-Rios Z., Overy D.P., Snowdon S., Nicholas J., Talbot N.J., Draper J. (2009). Metabolomic analysis reveals a common pattern of metabolic re-programming during invasion of three host plant species by *Magnaporthe grisea*. Plant J..

[42] Pan J.J., Clay K. (2003). Infection by the systemic fungus *Epichloe glyceriae* alters clonal growth of its grass host, *Glyceria striata*. Proc. R. Soc. Lond. B.

[43] Engelmann G. (1859). Two new dioecious grasses of the United States.

[44] Thompson B.E., Hake S. (2009). Translational biology: From Arabidopsis flowers to grass inflorescence architecture. Plant Physiol..

[45] Pautler M., Tanak W., Hirano H., Jackson D. (2013). Grass meristems I: Shoot apical meristem maintenance, axillary meristem determinacy and the floral transition. Plant Cell Physiol..

[46] Tanak W., Pautler M., Jackson D., Hirano H. (2013). Grass meristems II: Inflorescence architecture, flower development and meristem fate. Plant Cell Physiol..

[47] Chuck G., Meeley R., Irish E., Sakai H., Hake S. (2007). The maize tasselseed4 microRNA controls sex determination and meristem cell fate by targeting Tasselseed6/indeterminate spikelet1. Nat. Genet..

[48] Curaba J., Talbot M., Li Z., Helliwell C.C. (2013). Over-expression of microRNA171 affects phase transitions and floral meristem determinancy in barley. BMC Plant Biol..

[49] Huff D.R., Zagory D., Wu L. (1987). Report of buffalograss bunt (*Tilletia buchloëana*) in Oklahoma. Plant Dis..

[50] Huff D.R., Peakall R., Smouse P.E. (1993). RAPD variation within and among natural populations of outcrossing buffalograss. Theor. Appl. Genet..

[51] (2005). Minitab.

